# Comparative Politics, Political Settlements, and the Political Economy of Health Financing Reform: Comment on "Health Coverage and Financial Protection in Uganda: A Political Economy Perspective"

**DOI:** 10.34172/ijhpm.2023.7630

**Published:** 2023-02-26

**Authors:** Kevin Croke

**Affiliations:** Harvard School of Public Health, Boston, MA, USA.

**Keywords:** Health Financing Reform, Uganda, Stakeholder Analysis, Political Economy

## Abstract

Nannini et al analyze barriers to national health insurance reforms in Uganda using a political economy approach primarily rooted in stakeholder analysis. This approach is valuable, not only for its clear description of the interest-based politics at play, but also for its extension of stakeholder analysis to include consideration of the role of ideas and institutions in the policy process. However this analysis, and others like it, could be further strengthened by adding insights from two different sources. The first is the comparative politics literature on the Ugandan regime. The second is a related approach which analyzes public service delivery in the context of a country’s underlying "political settlement." Stakeholder-based approaches to health financing reform emphasize interest group conflict about the contents of policy reforms. By contrast, these complementary approaches imply distinct barriers to successful implementation of national health insurance in Uganda, rooted in the regime’s de-industrialization and the personalization of politics and resource allocation. They also suggest possible leverage points or avenues for progress which differ from those suggested by stakeholder analysis.

 Health systems pursue multiple goals: they seek to improve the health of those that use them, but also to protect users from falling into poverty. The paper by Nannini et al^1^ highlights the persistence of high out of pocket expenditures and catastrophic health expenditures in Uganda – 20 years after the removal of user fees in public sector health facilities – and the slow progress towards universal health coverage (UHC) via a proposed national health insurance program.

 The authors trace the dynamics of health financing in Uganda over a 20 year period, noting that low government health spending and persistently high out of pocket expenditures led health policy stakeholders to consider creating a national health insurance system. After more than a decade of advocacy and various policy proposals, a national health insurance bill was passed by parliament in 2021. However the authors are not optimistic that it will result in rapid progress towards a national insurance program; they conclude that “dominant interests and ideologies do not create a net incentive to implement a comprehensive scheme for this purpose.”

 The authors focus on ideas, institutions, and interests, paying particular attention to the interests andideas of key stakeholders. This approach shares much in common with other approaches to the politics of health financing reform.^2^ These approaches draw on an extensive set of cases to describe common dynamics of UHC-focused health financing reforms. In this framework, health financing is understood primarily as a question of distributional politics. Since national health insurance programs involve redistribution (through taxation and spending), reform entails conflict between winners and losers. Businesses and high-income individuals foresee higher taxes and labor costs, some formal sector workers fear that their existing benefits will be diluted, and doctors fear price controls or limits to their autonomy. The ostensible winners from national health insurance – the uninsured population – are often not mobilized around the issue. Instead, the actors in favor of reform can include social movements, non-governmental organisations, and activists representing poor and marginalized communities, as well as reformist health experts and policy-makers. At times political parties may take up the cause as an electoral tactic. Within government, the Ministry of Finance often opposes new spending programs, while other government agencies which run legacy insurance programs may resist reform or consolidation into a single national program. In low- and middle-income countries, donors and other international actors may play an important role. Unforeseen events such as economic crises, landslide electoral victories, or even natural disasters can provider unexpected “windows of opportunity” which disrupt existing coalitions and make progress possible.

 The authors’ analysis largely follows this model, by identifying stakeholders and conducting key informant interviews to assess their positions on national health insurance. They also usefully augment this model, by emphasizing the independent role that ideas and ideology can play in the process. On this basis they identify the government of Uganda’s support for a liberal, private sector-led development model as the key *ideological* factor hindering the adoption and implementation of a subsidized national health insurance program. They highlight the opposition of private sector firms, and of labor unions, as the central *interests* opposed to social health insurance. In their account, private sector insurance firms do not want to face competition from public health insurance, while unions fear increased mandatory salary deductions for their members who already benefit from health insurance. Among other stakeholders, academia and civil society are marginalized from policy making processes. Donors fund verticalized programs and do not play a strategic role on health policy.

 Interestingly, Nannini and colleagues’ analysis goes beyond a narrow stakeholder or interest group politics-based approach by emphasizing the roles of ideas, specifically the market-oriented approach of Uganda’s current leadership. By highlighting the ways in which the interest groups opposed to national health insurance gained influence through their alignment with the regime’s ideas about the role of the private sector in health, the authors provide a multi-faceted account of the slow progress of these reforms.

 Yet while this approach is a powerful framework for understanding health financing reforms in most cases, in this instance there are some additional considerations that could deepen their analysis. There are two ways that the authors might consider enriching it, to better capture important dynamics of the Uganda case.

 A first step would be to embed their political analysis in the findings of the political science literature on the current Ugandan regime. This research, typically found in the political science subfield of comparative politics, seeks to generate detailed, theoretically-informed accounts of politics in a given country. Topics includes the patterns and drivers of distributive politics, social service delivery, electoral coalitions, and state capacity. These analyses typically do not focus on the health sector per se, but the fundamental patterns that they describe are often visible in the operation of the health sector and health policy-making.

 Turning to this literature, recent studies of Ugandan politics share a key conclusion: Uganda under President Museveni has become a highly personalized regime. Rubongoya for example describes “deepening neopatrimonialism”^3^; similar accounts are found in Tripp,^4^ Kobusingye,^5^ and Tapscott.^6^ Personalist, neopatrimonial regimes are characterized by discretionary use of public resources to maintain coalitions of regime support, and the importance of personal relationships with an individual leader, rather than impersonal bureaucratic and legal procedures. While all political systems have elements of personalist politics, they become more prevalent in the event of prolonged rule by a single individual; in Uganda President Museveni has ruled since 1986. When these dynamics overtake regimes, adequate service delivery through the public sector becomes extremely challenging.

 With regard to health spending, Rugonboya,^3^ Kobusingye^5^ and Epstein^7^ highlight that an important reason for underspending on health in Uganda is that budgetary allocations flow preferentially to security forces and agencies linked to the presidency, while clientelist networks are further funded by the frequent creation of new districts, cabinet posts, and other public agencies. These dynamics from political science research are reinforced by Ugandan journalistic accounts.^8,9^ Donors fund a significant portion of health spending; however their protests at diversion of government resources from health are undermined by extensive security-sector aid from the same governments to Uganda, notably the United States.^7^

 A second, related approach, which would also strengthen a generic stakeholder analysis of health policy, is the concept of the “political settlement.” This concept is defined by Khan as “the ‘social order’ based on political compromises between powerful groups in society that sets the context for institutional and other policies.”^10^ The strength of this concept is that, like the country-specific political science literature, it can go beyond analysis of short run political coalitions in favor or against a specific policy proposal, by focusing on the deep structures of power in a given setting. The implications of Uganda’s political settlement for health in Uganda have been analyzed in recent work by Bukenya and Golooba-Mutebi,^11^ who seeks to explain regional variation in Uganda’s progress against maternal mortality. The authors use a rich theoretical framework and detailed comparative case studies to show the ways in which the basic political bargain among powerful actors which sustains the Uganda regime – the “political settlement” – deeply affects the delivery of health services. They analyze the political dynamics affecting national health politics, and the ways that these national dynamics can undermine health service delivery at the district level when local pro-development coalitions are not present. Their findings echo similar work on the relationship between high politics and key health priorities such as malaria control and other child health programs in Uganda.^12^

 These perspectives suggest different conclusions about the likely trajectory of national health insurance in Uganda. For example both approaches imply that normal interest group conflict might not be the major barrier to UHC. Interest group conflicts over program design can be overcome with new policy proposals. By contrast fundamental political bargains are less tractable. For example, the allocation of scarce public resources away from health and into other spending categories (State House, the military, district-level regime functionaries) suggests that the main opponents of major new investments in national health insurance might not be the private sector or medical associations, but rather state interests who benefit from current underspending on health.

 These approaches also highlight likely challenges in implementation. Implementation of national health insurance programs requires state capacity to collect premiums from a large informal sector, strategically purchase health services, and regulate private sector providers. A major theme of the political science literature on personalist, deinstitutionalized regimes is their strong tendency to undermine state capacity.

 These approaches also offer differing insights about possible paths forward. Analysts of the Ugandan political settlement note that Museveni’s regime has protected parts of the state – largely agencies seen as key to economic growth and regime stability, such as the Bank of Uganda, the Uganda Revenue Authority, and the Ministry of Finance, Planning and Economic Development – from clientelist pressure.^13^ One path towards creation of a revitalized public health sector might be an effort to convince the president that the health sector is strategically important in the same way that these economic agencies are.

 Alternatively, while state capacity has declined over time, Uganda’s economic growth, rising education levels, and semi-open political sphere have fostered an active press and growing civil society activism. The ability of these groups to drive policy towards UHC is limited in the short run, but support for their efforts may pay off when political circumstances change and a new window of opportunity for UHC opens up. Such approaches face long odds, but they are working “with the grain”^14^ – that is, in line with the fundamental political settlement – rather than at cross purposes to it.

 These analytical approaches need not be in tension with those used by Nannini et al rather they can be complementary. Analyzing how a country’s political settlement affects health has much in common with approaches which seek to identify the issue positions of the most powerful health sector actors. Similarly, since the authors have added analysis of ideas as well as institutions to their description of interest group politics, there is significant overlap between their approach and the comparative politics literature which also focuses heavily on the interplay between regime dynamics and state capacity.

## Ethical issues

 Not applicable.

## Competing interests

 Author declares that he has no competing interests.

## Author’s contribution

 KC is the single author of the paper.

## References

[1] Nannini M, Biggeri M, Putoto G (2021). Health coverage and financial protection in Uganda: a political economy perspective. Int J Health Policy Manag.

[2] Sparkes SP, Bump JB, Özçelik EA, Kutzin J, Reich MR (2019). Political economy analysis for health financing reform. Health Syst Reform.

[3] Rubongoya JB. Regime Hegemony in Museveni’s Uganda: Pax Musevenica. New York: Palgrave Macmillan; 2007.

[4] Tripp AM. Museveni’s Uganda: Paradoxes of Power in a Hybrid Regime. Boulder, CO: Lynne Rienner; 2010.

[5] Kobusingye O. The Patient: Sacrifice, Genius, and Greed in Uganda’s Healthcare System. AuthorHouse; 2019.

[6] Tapscott R. Arbitrary States: Social Control and Modern Authoritarianism in Museveni’s Uganda. New York: Oxford University Press; 2021.

[7] Epstein HC. Another Fine Mess: America, Uganda, and the War on Terror. Columbia Global Reports; 2017.

[8] Mwenda A. Understanding Museveni’s Grip on Uganda. The Independent; 2010.

[9] Mwenda A. Inside Museveni’s Political Game. The Independent; 2010.

[10] Khan M. Political Settlements and the Governance of Growth-Enhancing Institutions. Working Paper. 2010. https://eprints.soas.ac.uk/9968/1/Political_Settlements_internet.pdf. Accessed January 12, 2023.

[11] Bukenya B, Golooba-Mutebi F (2020). What explains sub-national variation in maternal mortality rates within developing countries? A political economy explanation. Soc Sci Med.

[12] Croke K (2012). The political economy of child mortality decline in Tanzania and Uganda, 1995-2007. Stud Comp Int Dev.

[13] Hickey S, Bukenya B, Matsiko H. Pockets of Effectiveness, Political Settlements and Technopols in Uganda: From State-Building to Regime Survival. 2021. ESID Working Paper No. 172. Manchester: Effective States and Inclusive Development Research Centre, The University of Manchester. https://www.effective-states.org/working-paper-172/. Accessed January 12, 2023.

[14] Levy B. Working with the Grain: Integrating Governance and Growth in Development Strategies. Oxford University Press; 2014.

